# Evaluation of Experienced Manipulation Characteristics in Robotic Surgery Using Log Data From the Hinotori Surgical System

**DOI:** 10.1002/ags3.70189

**Published:** 2026-01-30

**Authors:** Masaki Saito, Shunsuke Hayakawa, Hiroyuki Sagawa, Tamiaki Kondo, Masanao Ohashi, Sunao Ito, Reo Sato, Hajime Ushigome, Kenta Saito, Ryo Ogawa, Hiroki Takahashi, Shuji Takiguchi

**Affiliations:** ^1^ Graduate School of Medical Sciences Gastroenterological Surgery Nagoya City University Nagoya Japan; ^2^ Sysmex Corporation Kobe Japan

**Keywords:** hinotori, log data, manipulation, robotic surgery, surgical education

## Abstract

**Aim:**

This study aimed to examine characteristics of manipulation performed by experienced surgeons in robotic surgery by analyzing log data obtained from the Japanese surgical robot hinotori.

**Methods:**

Twelve gastrointestinal surgeons performed four tasks (transportation, dissection, single suturing, and continuous suturing), four times each. In total, 24 trials performed by six experienced surgeons were classified as the experienced (E) group, and 24 trials conducted by six less‐experienced surgeons were classified as the less‐experienced (L) group. Forceps log data were collected using the Medicaroid Intelligent Network, a hinotori network support system. Data on parameters such as instrument travel distance, velocity, acceleration, jerk, normalized mean squared jerk, wrist articulation counts, and scope manipulation characteristics were extracted and compared between the E and L groups.

**Results:**

In all tasks, the E group had significantly shorter task durations than the L group. The E group also exhibited higher average velocity, acceleration, jerk, and normalized mean squared jerk values in several tasks, particularly showing shorter travel distances with the right instrument (Arm3) and faster, more dynamically modulated movements with the left instrument (Arm1). Further, compared with the L group, the E group had a significantly longer camera movement per scope pedal press and significantly fewer wrist articulations in the suturing tasks.

**Conclusions:**

Experienced manipulation in robotic surgery is characterized by shorter instrument travel distances, intentional modulation of movement speed, and minimal wrist articulation. Quantitative evaluation of operative characteristics using log data can contribute to future applications in surgical training and skill assessment.

## Graphical abstract

This study analyzed log data from the Japanese hinotori surgical robot to characterize manipulation performed by experienced surgeons in robotic surgery. Compared with less‐experienced surgeons, the experienced group demonstrated shorter task durations, reduced travel distances with the right instrument (Arm3), faster and more dynamically modulated movements with the left instrument (Arm1), and markedly fewer unnecessary wrist articulations. They also operated the camera more efficiently, achieving a greater scope movement distance per pedal press. These findings highlight the potential of quantitative log data analysis for objective skill assessment and training in robotic surgery.

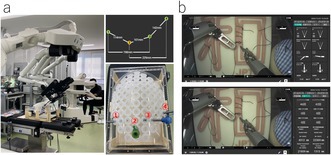

## 1 Introduction

In recent years, the widespread adoption of robot‐assisted surgery has led to a steady increase in the number of procedures performed. As this trend continues, the training of surgeons with advanced technical skills has become increasingly important. Thus, the development of efficient educational and training systems is considered a pressing issue. In the field of laparoscopic surgery, numerous studies have shown that training programs that incorporate checklists and organ models are effective [1, 2, 3, 4]. However, these approaches often involve substantial costs for model acquisition and require significant training time. Moreover, they primarily focus on preoperative preparation—such as gaining experience, understanding anatomy, and learning surgical procedures—rather than on refining the technical aspects of surgical manipulation itself.

In robot‐assisted surgery, simulators that can be used for practicing surgical techniques before performing them on actual patients are available. However, these simulators are extremely expensive, and typically only one trainee can use the system at a time. Consequently, there are still numerous challenges in providing practical and accessible education in robotic surgery.

To facilitate a faster and more efficient skill acquisition, it is important to identify and visualize the “key techniques” naturally used by experienced surgeons and to integrate them into training. However, these techniques are often challenging to articulate, and many are performed unconsciously by surgeons themselves, thereby making it challenging to incorporate them into structured educational frameworks.

The hinotori surgical robot, a domestically developed system, was launched in Japan in August 2020, and it has already been introduced into clinical practice at over 70 facilities. It is currently being utilized in various procedures across urology, gynecology, and gastrointestinal surgery [5, 6, 7, 8]. One of its key features is the ability to collect log data based on the coordinate positions of the tips of the robotic instruments. By analyzing these log data, the operational characteristics of individual surgeons can be evaluated objectively. We hypothesized that a detailed analysis of these data could reveal manipulation styles and skill components that are specific to experienced surgeons—that is, the “key techniques” underlying surgical expertise.

To date, no studies have conducted a comprehensive analysis of the robotic surgical log data obtained from actual procedures. Therefore, the current study aimed to investigate and characterize the surgical manipulation patterns of experienced surgeons by analyzing hinotori log data collected during standardized tasks.

## 2 Methods

### 2.1 Participants

**FIGURE 1:**
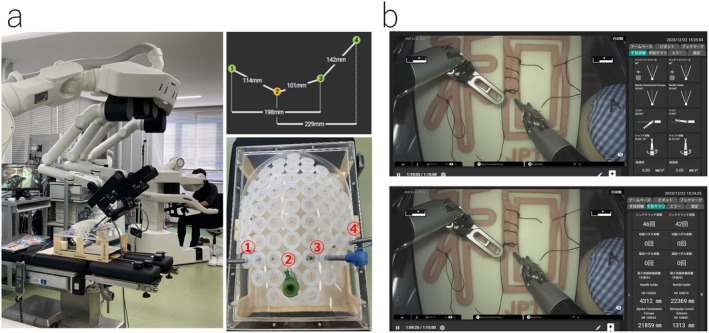
External view during task performance (a) and the Medicaroid Intelligent Network System (MINS) developed by Sysmex Corporation (b).

In total, 12 gastrointestinal surgeons participated in this study (Figure 1a). All participants were certified in robot‐assisted surgery. Six of them had performed > 50 robotic procedures and were classified as experienced surgeons. Meanwhile, the remaining six had performed ≤ 10 procedures and were considered less‐experienced surgeons. A brief background of the surgeons is provided in Table S1. Among the experienced surgeons, one specialized in esophageal surgery, two in gastric surgery, one in hepatobiliary‐pancreatic surgery, and two in colorectal surgery. Each of the 12 surgeons performed the four standardized tasks described below, four times each. The 24 trials performed by the six experienced surgeons were classified as the experienced group (E group), and the 24 trials performed by the six less‐experienced surgeons were designated as the less‐experienced group (L group). The log data obtained from these trials were then compared between the two groups. The ethics review committee of Nagoya City University approved this study (approval no: 60‐23‐0011).

### 2.2 Log Data Collection

In this study, surgical data (including operational logs from hinotori) were collected using the Medicaroid Intelligent Network (MINS), a network support system integrated with the hinotori surgical robot. Motion log data were generated by the Operation Unit of the hinotori Surgical Robot System and recorded during each task. These log data were utilized as part of a collaborative research project between Nagoya City University and Sysmex Corporation. In particular, data such as the surgeon's manipulation logs, task success rates, procedure durations, and error occurrences were obtained and used for analysis. This study was conducted as a collaborative research project with Sysmex Corporation, which provided financial support and technical assistance.

### 2.3 Robotic System and Setup

Log data were collected using the hinotori surgical robot. As shown in Figure 1a, an abdominal model manufactured by Fasotec was used for the procedures. The anatomical configuration was set to the pelvic region, and the hinotori was rolled in from the left side. The distance between the trocar pivot points was adjusted to 11–10–14 cm, with an acceptable error margin of < 0.5 cm. Although the hinotori system includes a hand control weight adjustment feature, the setting was fixed at a level 3 of 5. The motion scaling was set at 2:1.

The height of the armrest and the position of the foot pedals were adjusted according to the discretion of each surgeon. The surgeons were not informed of the task details in advance, and all instructions were provided via prerecorded audio guidance to eliminate variations in explanation.

The instruments varied based on the task. However, a 30° angled scope (Storz) was used for Arm 2 (A2) in all tasks. The robotic arms were defined as follows: Arm 1 (A1) for the left‐hand instrument, A2 for the camera, and Arm 3 (A3) for the right‐hand instrument. These three arms were included in the motion analysis. Arm 4 (A4), which was used for the assist instrument, was excluded from the analysis because of its limited usage time.

### 2.4 Collected Parameters

For each task, the following parameters were extracted using the Medicaroid Intelligent Network System (MINS). The MINS interface shown in Figure 1b is a prototype under development; however, it can measure and display values such as instrument opening angle, wrist angle, acceleration, the total number of hand‐clutch activations, and the total instrument travel distance.

In contrast, the three‐dimensional coordinates of each instrument tip were not displayed on the MINS interface and require a separate measurement system. Joint angles were measured using encoders integrated into the instrument housing of the hinotori surgical system. Based on the robotic kinematic model, the three‐dimensional coordinates of the instrument tip were calculated and recorded at 1‐s intervals. In addition, jerk had to be calculated separately using the formulas described later in the Methods section. The following data were collected and analyzed: task duration, the three‐dimensional coordinates of the tip of each instrument recorded at 1‐s intervals, the number of times the hand clutch was pressed, and the number of times the scope pedal was activated. In addition, for both the left and right instruments, the number of times the wrist articulation angle exceeded thresholds of 15°, 30°, and 40°, in either flexion or extension, was counted.

These data were collected to assess the frequency of wrist movements as an indicator of instrument articulation behavior.

### 2.5 Calculated Parameters Derived From the Collected Data

In this study, the following formulas were used to calculate the kinematic parameters. Here, t represents the duration of each task in seconds. The velocity, acceleration, and jerk were analyzed using their absolute values.

(1) Instrument Travel Distance (D: distance)

$$\sum_{i=1}^{t}\sqrt{\left(x_{i+1}-x_{i}\right)\;2+\left(y_{i+1}-y_{i}\right)\;2+\left(z_{i+1}-z_{i}\right)\;2}$$

(2) Average Speed

$$\frac{1}{t}\sum_{i=1}^{t}\sqrt{{\left(\frac{dx_{i}}{\mathrm{dt}}\right)}^{2}+{\left(\frac{dy_{i}}{\mathrm{dt}}\right)}^{2}+{\left(\frac{dz_{i}}{\mathrm{dt}}\right)}^{2}}$$

(3) Average Acceleration Magnitude

$$\frac{1}{t}\sum_{i=1}^{t}\sqrt{{\left(\frac{d^{2}x_{i}}{dt^{2}}\right)}^{2}+{\left(\frac{d^{2}y_{i}}{dt^{2}}\right)}^{2}+{\left(\frac{d^{2}z_{i}}{dt^{2}}\right)}^{2}}$$

(4) Average Jerk Magnitude (AJM)

$$\frac{1}{t}\sum_{i=1}^{t}\sqrt{{\left(\frac{d^{3}x_{i}}{dt^{3}}\right)}^{2}+{\left(\frac{d^{3}y_{i}}{dt^{3}}\right)}^{2}+{\left(\frac{d^{3}z_{i}}{dt^{3}}\right)}^{2}}$$

(5) Camera Movement Distance per Scope Pedal Activation.

The camera movement distance per scope pedal activation was calculated by dividing the total camera travel distance by the number of pedal activations.

(6) Normalized Mean Squared Jerk (NMSJ).

NMSJ has also been used for evaluating surgical skills and was calculated using the formula shown below [9]. Compared with AJM, NMSJ is less affected by task duration and movement distance.

$$\frac{1}{D^{2}\cdot t}\int_{0}^{t}{\left[j\left(t\right)\right]}^{2}\mathrm{d}t$$

(7) Supplementary explanation of each parameter.

To facilitate understanding, a conceptual diagram illustrating the relationships among Position, Velocity, Acceleration, and Jerk has been provided in Figure S1. Velocity represents the change in Position over time, Acceleration represents the change in Velocity over time, and Jerk represents the change in Acceleration over time. The absolute values of these quantities were used for the analyses.

In contrast, NMSJ is a dimensionless index calculated by dividing the squared jerk values by task duration and the square of the total travel distance, in order to remove the influence of these factors. This concept is similar to BMI, which divides body weight by height squared to minimize the effect of height. Therefore, NMSJ is more appropriately interpreted as a numerical value for comparison.

### 2.6 Task 1—Object Transportation Model

**FIGURE 2:**
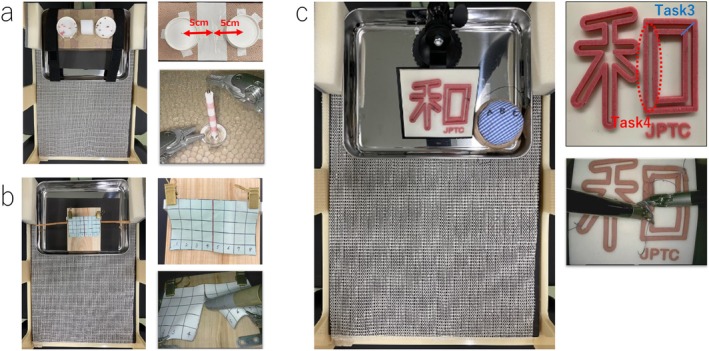
Overview of all tasks. (a) Task 1; (b) Task 2; (c) Task 3 and Task 4.

Task 1 involved transporting objects, and it was designed to identify the factors associated with a faster and more accurate grasping and transportation. Paper cups were placed upside down at 10‐cm intervals. Ten brown beads were placed in the left cup and ten pink beads in the right cup. A thumbtack was fixed upside down in the center of each paper cup, and the participants were instructed to thread the two colors of beads alternately onto the tack. The task was considered complete when four beads of each color (eight in total) were stacked in alternating order (Figure 2a).

Needle holders were used on both A1 and A3. A4 was not used in this task.

### 2.7 Task 2—Cutting Model

Task 2 involved a cutting motion and aimed to assess the factors associated with precise and efficient cutting. A commercially available 8 × 5 cm rubber sheet was used, with 1 × 1 cm grid lines drawn on its surface. The top corners were fixed at two points, and a 0.2 cm red guideline was drawn down the center. The rubber sheet was then cut along this line to divide it into two parts. The task was completed once the cut was finished (Figure 2b). The instruments used were bipolar fenestrated forceps on A1, monopolar curved scissors on A3, and a universal grasping forceps on A4.

### 2.8 Task 3—Single Suturing and Knot Tying

Task 3 aimed to evaluate the factors related to accurate and efficient single suturing and knot tying. The “Wa Model” (produced by JPTC Co. Ltd.) was used. Single suturing was performed at the upper right section of the model, followed by three knot ties. A 3‐0 Silk suture cut to a length of 10 cm was used. The suture was placed on the model platform at the beginning of the task, and the task commenced from the point of grasping the needle. The task was completed after three knot ties were performed (Figure 2c).

The instruments used were bipolar fenestrated forceps on A1, a needle holder on A3, and a universal grasping forceps on A4.

### 2.9 Task 4–Continuous Suturing and Knot Tying

Task 4 aimed to assess the factors associated with smooth and precise continuous suturing and knot tying. The same “Wa Model” used in task 3 (JPTC Co. Ltd.) was utilized. A 3‐0 Silk suture, cut to a length of 16 cm, was used. The suture was placed on the platform of the model at the beginning of the task, which began from the point of picking up the needle. One suture was placed on the central protrusion of the model, followed by three knot ties. Then, four continuous sutures were placed vertically from top to bottom, followed by another three knot ties (Figure 2c).

The same instruments as in task 3 were used: bipolar fenestrated forceps on A1, needle holder on A3, and universal grasping forceps on A4. Two instances of suture breakage occurred in the less‐experienced group during this task, and these cases were excluded from the analysis.

### 2.10 Statistical Analysis

Statistical analyses were performed using the statistical software EZR [10]. The Mann–Whitney *U* test was used to examine continuous variables. Consequently, the values were presented as the median and interquartile range (Q1 and Q3). A *p* value of < 0.05 indicated statistically significant differences.

## 3 Results

### 3.1 Task 1

Table 1 shows the results for task 1. The experienced group (E group) had a significantly shorter task duration and pressed the scope pedal fewer times than the less‐experienced group (L group). The instrument travel distance was significantly shorter in the E group than in the L group in both A1 and A3. The average speed for A1 and A3 was significantly higher in the E group than in the L group. The E group had a significantly greater average acceleration for A3 than the L group. There was no significant difference in terms of NMSJ between the two groups.

**TABLE 1:** Comparison between the experienced and less‐experienced groups for task 1 results.

	Experienced group (*n* = 24)	Less‐experienced group (*n* = 24)	*p*
Task duration (s)	85.5 (74–97)	126.5 (83.5–150.25)	0.001
Travel distance A1 (mm)	736.59 (650.9–834.3)	963.96 (767.06–1033.2)	0.003
Travel distance A2 (mm)	458.73 (374.2–570.2)	603.64 (409.81–672.57)	0.174
Travel distance A3 (mm)	789.57 (691.6–824.3)	925.31 (785.5–1003.1)	0.004
Mean speed A1 (mm/s)	8.93 (7.99–9.57)	7.33 (6.87–8.59)	0.005
Mean speed A2 (mm/s)	5.17 (4.52–6.33)	4.56 (3.65–5.52)	0.055
Mean speed A3 (mm/s)	8.79 (8.14–9.66)	7.17 (6.59–7.82)	0.001
Mean acceleration A1 (mm/s^2^)	7.36 (6.94–8.60)	6.64 (6.10–8.50)	0.303
Mean acceleration A3 (mm/s^2^)	7.65 (7.05–8.37)	6.29 (5.94–7.61)	0.020
AJM A1 (mm/s^3^)	12.69 (11.15–14.81)	12.06 (10.43–14.89)	0.550
AJM A3 (mm/s^3^)	13.14 (11.78–15.04)	11.13 (10.03–13.40)	0.066
NMSJ A1 (×10^−5^)	52.81 (31.24–60.95)	33.49 (19.91–64.80)	0.159
NMSJ A3 (×10^−5^)	51.06 (32.33–66.99)	27.41 (19.41–59.19)	0.065
Right‐hand clutch (times)	1 (0–2)	1 (0–2)	0.863
Left‐hand clutch (times)	1 (0–2)	1 (0–2.25)	0.231
Total hand clutch (times)	2 (0–4)	2 (1–5)	0.476
Scope pedal (times)	11.5 (7–16)	16 (10.50–20.25)	0.019
Scope movement per pedal (mm)	40.01 (36.67–47.16)	31.38 (27.47–35.57)	0.003
A1 articulation ≥ 15° (times)	12.00 (9.00–16.25)	15.00 (13.75–20.00)	0.008
A1 articulation ≥ 30° (times)	6.00 (4.00–10.00)	8.00 (4.50–12.00)	0.461
A1 articulation ≥ 40° (times)	2.00 (1.50–4.25)	3.00 (1.50–7.25)	0.508
A3 articulation ≥ 15° (times)	12.50 (10.00–15.25)	16.00 (12.00–19.25)	0.016
A3 articulation ≥ 30° (times)	6.00 (5.50–8.00)	6.00 (4.00–10.25)	0.950
A3 articulation ≥ 40° (times)	4.00 (2.00–6.00)	4.00 (2.00–6.00)	0.774

*Note:* Median and interquartile range (IQR: 25%–75%).

Abbreviations: A1, Arm 1 (left manipulator); A2, Arm 2 (camera); A3, Arm 3 (right manipulator); AJM, average jerk magnitude; NMSJ, normalized mean squared jerk.

The E group had a significantly longer camera movement distance per scope pedal activation than the L group. The number of wrist articulations at ≥ 15° in A1 was significantly lower in the E group than in the L group.

### 3.2 Task 2

Table 2 shows the results for task 2. The E group had a significantly shorter task duration than the L group. The instrument travel distance for A1 and A3 was significantly shorter in the E group than in the L group. The E group had a significantly higher average speed for A1 and A2 than the L group. The average acceleration and AJM for A3 were significantly greater in the E group than in the L group. The NMSJ was significantly higher in the E group than in the L group for both A1 and A3. The E group had a significantly longer camera movement distance per scope pedal activation than the L group. No significant differences were observed between the two groups in terms of the number of wrist articulations.

**TABLE 2:** Comparison between the experienced and less‐experienced groups for task 2 results.

	Experienced group (*n* = 24)	Less‐experienced group (*n* = 24)	*p*
Task duration (s)	61.5 (65.75–76.00)	92.5 (84.75–101.00)	< 0.001
Travel distance A1 (mm)	747.19 (631.26–853.80)	871.98 (768.20–1017.48)	0.005
Travel distance A2 (mm)	146.45 (111.48–175.42)	137.93 (117.08–157.04)	0.284
Travel distance A3 (mm)	553.81 (499.09–661.15)	758.34 (621.35–848.82)	0.001
Mean speed A1 (mm/s)	12.03 (10.88–13.15)	10.09 (8.72–10.86)	0.004
Mean speed A2 (mm/s)	2.32 (1.97–2.86)	1.37 (1.13–1.65)	< 0.001
Mean speed A3 (mm/s)	9.11 (8.44–9.72)	8.91 (6.12–9.84)	0.343
Mean acceleration A1 (mm/s^2^)	2.33 (1.81–3.19)	2.3 (1.69–3.19)	0.869
Mean accelerationA3 (mm/s^2^)	4.69 (4.06–5.15)	2.99 (2.44–3.56)	< 0.001
AJM A1 (mm/s^3^)	3.9 (2.90–5.26)	3.78 (2.43–5.32)	0.902
AJM A3 (mm/s^3^)	8.2 (7.42–9.01)	5.18 (4.06–6.28)	< 0.001
NMSJ A1 (×10^−5^)	278.01 (164.67–586.50)	139.89 (93.85–271.18)	0.020
NMSJ A3 (×10^−5^)	167.95 (97.86–261.85)	79.35 (31.95–121.53)	0.006
Right‐hand clutch (times)	1 (0.75–2.00)	1 (0–2.00)	0.455
Left‐hand clutch (times)	1 (0–1.25)	1 (0–2.00)	0.908
Total hand clutch (times)	1 (0.75–3.00)	1.5 (0–4.00)	0.303
Scope pedal (times)	2.5 (1.75–4.00)	3 (2.00–5.00)	0.098
Scope movement per pedal (mm)	55.22 (42.42–83.68)	30.94 (21.09–53.31)	0.004
A1 articulation ≥ 15° (times)	2.00 (0.00–11.00)	2.00 (0.00–8.00)	0.579
A1 articulation ≥ 30° (times)	0.50 (0.00–4.00)	0.00 (0.00–6.00)	0.141
A1 articulation ≥ 40° (times)	0.00 (0.00–4.00)	0.00 (0.00–2.00)	0.097
A3 articulation ≥ 15° (times)	2.00 (0.00–11.00)	3.00 (0.00–18.00)	0.731
A3 articulation ≥ 30° (times)	1.00 (0.00–8.00)	0.00 (0.00–6.00)	0.085
A3 articulation ≥ 40° (times)	0.00 (0.00–2.00)	0.00 (0.00–2.00)	0.899

*Note:* Median and interquartile range (IQR: 25%–75%).

Abbreviations: A1, Arm 1 (left manipulator); A2, Arm 2 (camera); A3, Arm 3 (right manipulator); AJM, average jerk magnitude; NMSJ, normalized mean squared jerk.

### 3.3 Task 3

Table 3 shows the results for task 3. The E group had a significantly shorter task duration than the L group. The instrument travel distance for A3 was significantly shorter in the E group than in the L group. The E group had a significantly higher average speed for A1 and A2 than the L group. The average acceleration and AJM for A1 and A3 were significantly greater in the E group than in the L group. The E group had a significantly higher NMSJ than the L group for both A1 and A3. The camera movement distance per scope pedal activation was significantly longer in the E group than in the L group. The number of wrist articulations was significantly lower in the E group than in the L group at ≥ 30° for A1, and at ≥ 15°, ≥ 30°, and ≥ 40° for A3.

**TABLE 3:** Comparison between the experienced and less‐experienced groups for task 3 results.

	Experienced group (*n* = 24)	Less‐experienced group (*n* = 24)	*p*
Task duration (s)	59.50 (51.00–68.75)	87.00 (69.75–102.75)	< 0.001
Travel distance A1 (mm)	648.12 (597.50–709.36)	677.31 (604.81–832.54)	0.232
Travel distance A2 (mm)	98.89 (68.61–135.19)	79.92 (55.43–95.40)	0.053
Travel distance A3 (mm)	555.66 (491.63–653.07)	646.91 (566.70–792.92)	0.013
Mean speed A1 (mm/s)	11.65 (9.86–12.75)	8.92 (7.53–10.02)	0.001
Mean speed A2 (mm/s)	1.68 (1.27–2.09)	1.03 (0.67–1.15)	< 0.001
Mean speed A3 (mm/s)	9.46 (8.52–10.48)	8.37 (6.81–9.94)	0.066
Mean acceleration A1 (mm/s^2^)	16.01 (13.25–19.01)	10.76 (9.99–14.89)	0.001
Mean acceleration A3 (mm/s^2^)	11.91 (11.08–13.22)	9.42 (8.37–12.17)	0.015
AJM A1 (mm/s^3^)	16.01 (13.25–19.01)	11.37 (9.99–14.89)	0.001
AJM A3 (mm/s^3^)	11.91 (11.08–13.22)	9.42 (8.37–12.17)	0.011
NMSJ A1 (×10^−5^)	84.64 (60.06–115.70)	32.10 (24.71–53.55)	< 0.001
NMSJ A3 (×10^−5^)	68.50 (42.64–117.81)	38.69 (25.14–47.89)	0.002
Right‐hand clutch (times)	0.00 (0.00–1.00)	0.5 (0.00–1.00)	0.457
Left‐hand clutch (times)	0.00 (0.00–1.00)	0 (0.00–2.00)	0.763
Total hand clutch (times)	0.50 (0.00–2.00)	0.5 (0.00–3.00)	0.595
Scope pedal (times)	2.00 (1.00–3.00)	3 (1.00–4.00)	0.203
Scope movement per pedal (mm)	47.79 (26.67–70.35)	27.17 (19.90–46.34)	0.050
A1 articulation ≥ 15° (times)	8.00 (5.75–12.75)	11 (8.75–14.25)	0.086
A1 articulation ≥ 30° (times)	2.00 (2.00–4.00)	7 (3.50–9.50)	0.005
A1 articulation ≥ 40° (times)	0.50 (0.00–2.00)	2 (0.00–8.00)	0.38
A3 articulation ≥ 15° (times)	15.00 (12.00–17.25)	17.5 (15.00–22.00)	0.005
A3 articulation ≥ 30° (times)	6.00 (4.00–8.50)	10.5 (7.25–14.50)	0.007
A3 articulation ≥ 40° (times)	2.00 (0.00–4.00)	6 (4.00–9.00)	< 0.001

*Note:* Median and interquartile range (IQR: 25%–75%).

Abbreviations: A1, Arm 1 (left manipulator); A2, Arm 2 (camera); A3, Arm 3 (right manipulator); AJM, average jerk magnitude; NMSJ, normalized mean squared jerk.

### 3.4 Task 4

Table 4 shows the results for task 4. The task duration was significantly shorter in the E group than in the L group. The E group had a significantly shorter instrument travel distance for A2 and A3 than the L group. The average speed for A1 and A2 was significantly higher in the E group than in the L group. The average acceleration and AJM for A1 were significantly greater in the E group than in the L group. The NMSJ for A1 was also significantly higher in the E group than in the L group. The number of wrist articulations was significantly lower in the E group than in the L group at ≥ 30° and ≥ 40° for A1 and at ≥ 15°, ≥ 30°, and ≥ 40° for A3. Figure S2 shows the angular changes in the wrist articulation.

**TABLE 4:** Comparison between the experienced and less‐experienced group for task 4 results.

	Experienced group (*n* = 24)	Less‐experienced group (*n* = 22)	*p*
Task duration (s)	221.5 (196.26–235.50)	290 (249.25–328.25)	0.001
Travel distance A1 (mm)	2159.44 (2025.90–2640.26)	2507.19 (2275.68–2850.78)	0.095
Travel distance A2 (mm)	248.48 (121.37–397.10)	136.51 (108.91–210.54)	0.031
Travel distance A3 (mm)	1743.04 (1508.23–2214.60)	2104.64 (1965.52–2417.45)	0.002
Mean speed A1 (mm/s)	11.4 (9.71–12.13)	9.17 (7.91–10.58)	0.006
Mean speed A2 (mm/s)	1.33 (0.60–1.76)	0.52 (0.36–0.73)	< 0.001
Mean speed A3 (mm/s)	9.09 (6.42–9.61)	8.1 (7.52–9.04)	0.538
Mean acceleration A1 (mm/s^2^)	8.5 (7.84–9.29)	7.57 (6.43–8.54)	0.01
Mean accelerationA3 (mm/s^2^)	6.06 (4.79–7.28)	6.22 (4.90–6.57)	0.809
AJM A1 (mm/s^3^)	14.72 (13.76–16.22)	13.21 (11.39–14.52)	0.017
AJM A3 (mm/s^3^)	11.18 (8.81–11.98)	10.32 (8.78–12.40)	0.553
NMSJ A1 (×10^−5^)	6.05 (4.78–8.33)	4.23 (3.23–6.93)	0.048
NMSJ A3 (×10^−5^)	5.53 (3.19–7.67)	3.71 (2.68–6.73)	0.200
Right‐hand clutch (times)	2.00 (1.00–3.00)	1.00 (1.00–3.75)	0.604
Left‐hand clutch (times)	1.00 (0.75–2.00)	1.50 (0.25–2.00)	0.627
Total hand clutch (times)	3.00 (2.0–4.25)	3.00 (1.25–6.75)	0.542
Scope pedal (times)	8 (3.75–13.0)	5 (3.00–10.00)	0.116
Scope movement per pedal (mm)	32.42 (27.55–41.07)	27.92 (19.83–44.53)	0.322
A1 articulation ≥ 15° (times)	33 (22.00–39.25)	39.5 (26.50–48.75)	0.055
A1 articulation ≥ 30° (times)	15 (9.50–31.50)	24 (17.75–33.75)	0.045
A1 articulation ≥ 40° (times)	6 (2.00–12.00)	13 (6.00–19.50)	0.05
A3 articulation ≥ 15° (times)	46.5 (37.75–54.50)	57.5 (51.25–65.00)	0.009
A3 articulation ≥ 30° (times)	16 (7.50–23.00)	29 (24.50–42.25)	0.001
A3 articulation ≥ 40° (times)	3 (1.50–10.00)	16.5 (6.25–22.75)	< 0.001

*Note:* Median and interquartile range (IQR: 25%–75%).

Abbreviations: A1, Arm 1 (left manipulator); A2, Arm 2 (camera); A3, Arm 3 (right manipulator); AJM, average jerk magnitude; NMSJ, normalized mean squared jerk.

### 3.5 Summary of Findings

**FIGURE 3:**
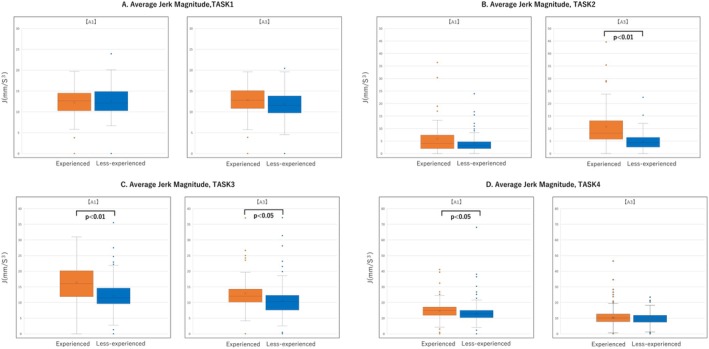
Comparison of average jerk magnitude (AJM) across all tasks.

In all tasks, the E group had significantly shorter task durations than the L group. The number of scope pedal activations was higher in the E group than in the L group in one task. The instrument travel distance was shorter in the E group than in the L group for A3 across all tasks, for A1 in two tasks, and for A2 in one task. The average instrument speed was higher in the E group than in the L group for A1 in all tasks, for A2 in three tasks, and for A3 in one task. The average acceleration was significantly greater in the E group than in the L group for A3 in three tasks and for A1 in two tasks (Figure S3). The AJM was significantly greater in the E group than in the L group for A1 and A3 in two tasks (Figure 3). The E group had a significantly higher NMSJ than the L group for A1 in three tasks and for A3 in two tasks. The camera movement distance per scope pedal activation was significantly longer in the E group than in the L group in three tasks. For A1, the E group had significantly fewer wrist articulations ≥ 15° in one task, ≥ 30° in two tasks. Similarly, for A3, the E group had significantly fewer articulations at ≥ 15° in three tasks, ≥ 30° and ≥ 40° in two tasks.

Table 5 shows the summary of all tasks. Significant differences observed in two or more tasks were as follows:

**TABLE 5:** Number of tasks showing significant differences for each arm.

	Number of tasks with a significant difference Arm 1	Number of tasks with a significant difference Arm 2	Number of tasks with a significant difference Arm 3
Travel distance	2	1	4
Mean speed	4	3	1
Mean acceleration	2	—	3
AJM	2	—	2
NMSJ	3	—	2
Scope movement per pedal	—	3	—
Articulation ≥ 15°	1	—	3
Articulation ≥ 30°	2	—	2
Articulation ≥ 40°	0	—	2

Abbreviations: A1, Arm 1 (left manipulator); A2, Arm 2 (camera); A3, Arm 3 (right manipulator); AJM, average jerk magnitude; NMSJ, normalized mean squared jerk.

For Arm 1, the parameters were travel distance, mean speed, mean acceleration, AJM, NMSJ, and articulation ≥ 30°.mFor Arm 2, the parameters were mean speed and scope movement per pedal. For Arm 3, the parameters were travel distance, mean acceleration, AJM, NMSJ, and articulations ≥ 15°, ≥ 30°, and ≥ 40°. To further facilitate readers' understanding, we created subtitled video clips demonstrating typical movements of surgeons in the E group and L group for each task (Videos S1–, S4).

## 4 Discussion

This study is the first to analyze robotic surgical operations using log data. The E group had significantly shorter task durations in all tasks. In several tasks, the E group also had shorter instrument travel distances and a higher average speed, acceleration, AJM, and NMSJ. Our results showed that experienced surgeons used the right hand more efficiently by taking the shortest optimal path and moved the left hand faster than those in the L group. The camera was also operated more efficiently and rapidly. In addition, the distance the camera traveled per scope pedal press was longer in the E group. Hence, the experienced surgeons were able to reposition the camera appropriately with fewer actions. No significant differences were observed in the number of instrument articulations between the E and L groups in tasks 1 and 2. However, in the suturing tasks (Tasks 3 and 4), the E group exhibited fewer articulations, indicating a more efficient wrist control with minimal unnecessary bending.

Based on these quantitative findings, we further analyzed the characteristics of each instrument arm and the camera. Mean acceleration, AJM, and NMSJ showed significant differences between the groups in multiple tasks for both the left and right hands, indicating that these parameters may serve as common and reliable indicators for differentiating skill levels. Mean speed appeared to be a more important parameter for the left hand, whereas travel distance and wrist articulation were more sensitive parameters for the right hand, showing clearer distinctions between experienced and less‐experienced surgeons. Furthermore, for the camera, a greater scope movement distance per pedal activation was identified as a characteristic indicator of experienced manipulation. These findings provide novel insights into robotic surgical technique. Further, this study is the first to evaluate robotic surgery‐specific metrics such as instrument articulation angles and scope movement distance per pedal activation, emphasizing the unique contributions of our analysis.

Previous studies using open surgical simulations have reported that experienced surgeons tend to have lower acceleration and jerk values [9, 11]. In contrast, similar studies conducted in laparoscopic simulation environments have found that experienced surgeons maintain shorter travel distances and faster speeds. However, they are also more likely to exhibit greater acceleration and jerk [12, 13, 14]. Further, other studies that analyzed instrument motion in actual gastrectomy procedures using artificial intelligence have reported no significant differences between experienced surgeons and less‐experienced surgeons in terms of speed, acceleration, or jerk. Similarly, a report on cholecystectomy found that experienced surgeons had a lower acceleration. Nevertheless, there was no difference in jerk between experienced surgeons and less‐experienced surgeons [15, 16]. These conflicting findings suggest that the association between expertise and motion dynamics remains controversial.

Flash and Hogan reported that in simple point‐to‐point movements, smooth trajectories that decrease jerk are considered indicators of skilled motor performance [17]. In contrast, our study found that experienced surgeons exhibited greater acceleration and jerk in a complex robotic surgical environment compared with less‐experienced surgeons. This may reflect not mere smoothness but the ability to intentionally regulate movement speed in response to task demands—that is, purposeful control of acceleration and deceleration. In advanced surgical tasks such as suturing, real‐time integration of visual and motor information is essential for precise and prompt actions, which may manifest as higher jerk values. Thus, increased jerk should not be viewed solely as a sign of inexperience. Rather, it may represent a hallmark of an advanced and efficient motion strategy. This concept is not challenging to imagine when considering examples such as motor sports, where applying strategic acceleration and deceleration during cornering leads to faster lap times. Based on these results, we propose that encouraging modulated, dynamic motion control, rather than emphasizing constant slow movements, may be a key to skill acquisition in robotic surgical training.

The greatest advantage of using log data in robotic surgery is its ability to capture precise intra‐abdominal instrument movement, unlike external motion capture systems. This allows for the accurate and practical analysis of real surgical behavior and enables objective motion evaluation. Moreover, this study first performed a quantitative examination of robotic‐specific operations such as instrument articulation angles and scope movement distance per foot pedal activation. Continued research in this area may contribute to the more rapid acquisition of technical skills among surgeons. These findings also provide the foundation for developing a quantitative scoring system that can objectively assess surgical skill and quality in robotic surgery in future studies. Further, such findings could ultimately support the development of autonomous or semi‐autonomous surgical technologies.

This study has three main limitations. First, it was conducted in a dry‐lab environment, and the instrument motion in such settings may differ from real surgical conditions. In addition, further validation using wet models, such as fresh porcine organs, or 3D training models for lymph node dissection may help to enhance the clinical significance of this preclinical study. These approaches will be considered in future research. Future studies should analyze actual clinical performance using this system, as log data can also be obtained during real procedures. We believe that clinical research utilizing this approach will continue to advance. Second, the relatively small number of participants and the limited number of trials may restrict the generalizability of the findings. This limitation was mainly due to the logistical constraints of the experimental setting. Each surgeon spent an entire day performing multiple tasks, and the hinotori had to be exclusively reserved for these experiments. Therefore, the number of participants and repetitions was restricted by time, budgetary, and equipment availability constraints. Third, as this is the first study to use robotic surgical log data, the suitability of the task designs used in this study has not been completely validated. Thus, future investigations involving multiple institutions should be performed to refine task selection and collect additional data for broader validation.

In conclusion, experienced manipulation in robot‐assisted surgery is characterized by shorter travel distances and dynamically modulated motion, resulting in an efficient and precise performance with minimal unnecessary motion. The quantitative evaluation of motion characteristics using log data is significantly promising for future surgical education, skill assessment, and the advancement of surgical autonomous or semi‐autonomous technologies.

## Author Contributions

**Masaki Saito:** methodology, writing – original draft, visualization, writing – review and editing, data curation, formal analysis, project administration. **Shunsuke Hayakawa:** conceptualization, methodology, formal analysis, visualization, project administration, funding acquisition, supervision, data curation, validation. **Hiroyuki Sagawa:** investigation, supervision. **Tamiaki Kondo:** data curation, software, resources. **Masanao Ohashi:** software, data curation, resources. **Sunao Ito:** writing – review and editing. **Reo Sato:** writing – review and editing. **Hajime Ushigome:** investigation. **Kenta Saito:** data curation, writing – review and editing. **Ryo Ogawa:** investigation. **Hiroki Takahashi:** investigation, validation. **Shuji Takiguchi:** funding acquisition, writing – review and editing.

## Funding

This study was conducted as a collaborative research project with Sysmex Corporation, which provided financial support and technical assistance.

## Ethics Statement

The study was approved by the Institutional Review Board of the Graduate School of Medical Sciences, Nagoya City University. The approval number is 60‐23‐0011.

## Consent

The authors have nothing to report.

## Conflicts of Interest

This study was conducted as part of a collaborative research project between Nagoya City University, Graduate School of Medical Sciences, Gastroenterological Surgery, and Sysmex Corporation. Log data acquisition was supported by the company; however, the company had no role in study design, manuscript preparation, or publication decisions. Shuji Takiguchi is an editorial board member of Annals of Gastroenterological Surgery. The other authors declare no conflicts of interest.

## Supporting information


**Figure S1:** A conceptual diagram illustrating the relationships among Position, Velocity, Acceleration, and Jerk.

**Figure S2:** Angular changes in wrist articulation during Task 4.

**Figure S3:** Comparison of average acceleration across all tasks.

**Table S1:** Background characteristics of E and L groups.

**Video S1:** This video presents characteristic motion segments of the E and L groups in Task 1. The E group demonstrates appropriate wrist articulation, minimal and efficient scope adjustments, and fast, straight instrument trajectories toward the target.

**Video S2:** This video presents characteristic motion segments of the E and L groups in Task 2. The E group performs dissection with appropriate tension, shows dynamic yet well‐controlled instrument movement, and utilizes wide, accurate scope adjustments. The instrument follows a fast, relatively direct, and efficient path toward the target.

**Video S3:** This video presents characteristic motion segments of the E and L groups in Task 3. The instrument moves quickly along a nearly shortest path, with wrist articulations occurring only when appropriate. The scope remains properly positioned, and the instrument demonstrates dynamic yet well‐controlled movement.

**Video S4:** This video presents characteristic motion segments of the E and L groups in Task 4. The left instrument moves quickly with dynamic yet controlled variation, while the right instrument follows a direct path. Wrist articulations occur only at appropriate moments throughout the procedure.

## References

[1] S. Poudel , Y. Kurashima , Y. Kawarada , et al., “Development and Validation of a Checklist for Assessing Recorded Performance of Laparoscopic Inguinal Hernia Repair,” American Journal of Surgery 212, no. 3 (2016): 468–474. 26750607 10.1016/j.amjsurg.2015.09.014

[2] M. Thomaschewski , R. Vonthein , T. Keck , T. Laubert , and C. Benecke , “Laparoscopic Simulation Training Improves Operating Room Performance of Surgical Residents: A Multicenter Randomized Trial (NOVICE),” International Journal of Surgery 111, no. 4 (2025): 2923–2932. 39998559 10.1097/JS9.0000000000002304 PMC12175799

[3] S. Poudel , Y. Kurashima , K. Tanaka , et al., “Educational System Based on the TAPP Checklist Improves the Performance of Novices: A Multicenter Randomized Trial,” Surgical Endoscopy 32, no. 5 (2018): 2480–2487. 29124407 10.1007/s00464-017-5950-x

[4] A. Shibuya , Y. Isobe , Y. Nishihara , S. Matsumoto , T. Nagayasu , and K. Matsumoto , “Development and Validation of a High‐Quality Simulator With Exchangeable Peritoneum for Transabdominal Preperitoneal Laparoscopic Inguinal Hernia Repair,” Asian Journal of Endoscopic Surgery 17, no. 4 (2024): e13362. 39045770 10.1111/ases.13362

[5] H. Noshiro , T. Ide , A. Nomura , Y. Yoda , M. Hiraki , and T. Manabe , “Introduction of a New Surgical Robot Platform “Hinotori” in an Institution With Established da Vinci Surgery for Digestive Organ Operations,” Surgical Endoscopy 38, no. 7 (2024): 3929–3939. 38839604 10.1007/s00464-024-10918-4

[6] S. Togami , T. Higashi , A. Tokudome , et al., “The First Report of Surgery for Gynecological Diseases Using the Hinotori Surgical Robot System,” Japanese Journal of Clinical Oncology 53, no. 11 (2023): 1034–1037. 37595992 10.1093/jjco/hyad105

[7] D. Motoyama , Y. Matsushita , H. Watanabe , et al., “Robot‐Assisted Radical Nephrectomy Using Novel Surgical Robot Platform, Hinotori: Report of Initial Series of 13 Cases,” International Journal of Urology 30, no. 12 (2023): 1175–1179. 37654155 10.1111/iju.15292

[8] H. Tsujioka , K. Setoguchi , A. Nirazuka , et al., “Comparison of Robot‐Assisted Laparoscopic Prostatectomy Using the Made‐In‐Japan Robotic System Hinotori Versus da Vinci: A Propensity Score‐Matched Analysis,” International Journal of Medical Robotics + Computer Assisted Surgery: MRCAS 20, no. 6 (2024): e70013. 39655698 10.1002/rcs.70013

[9] A. Ghasemloonia , Y. Maddahi , K. Zareinia , S. Lama , J. C. Dort , and G. R. Sutherland , “Surgical Skill Assessment Using Motion Quality and Smoothness,” Journal of Surgical Education 74, no. 2 (2017): 295–305. 27789192 10.1016/j.jsurg.2016.10.006

[10] Y. Kanda , “Investigation of the Freely Available Easy‐to‐Use Software ‘EZR’ for Medical Statistics,” Bone Marrow Transplantation 48, no. 3 (2013): 452–458. 23208313 10.1038/bmt.2012.244 PMC3590441

[11] P. Deepika , K. V. V. Deepesh , P. S. Vadali , M. Rao , V. Vazhayil , and A. M. Uppar , “Computer Assisted Objective Assessment of Micro‐Neurosurgical Skills From Intraoperative Videos,” IEEE Open Journal of Engineering in Medicine and Biology 4 (2023): 11–20. 37057038 10.1109/OJEMB.2023.3257987 PMC10089269

[12] K. Ebina , T. Abe , M. Higuchi , et al., “Motion Analysis for Better Understanding of Psychomotor Skills in Laparoscopy: Objective Assessment‐Based Simulation Training Using Animal Organs,” Surgical Endoscopy 35, no. 8 (2021): 4399–4416. 32909201 10.1007/s00464-020-07940-7 PMC8263434

[13] L. Yan , K. Ebina , T. Abe , et al., “Validation and Motion Analyses of Laparoscopic Radical Nephrectomy With Thiel‐Embalmed Cadavers,” Current Problems in Surgery 61, no. 10 (2024): 101559. 39266126 10.1016/j.cpsurg.2024.101559

[14] K. Ebina , T. Abe , K. Hotta , et al., “Automatic Assessment of Laparoscopic Surgical Skill Competence Based on Motion Metrics,” PLoS One 17, no. 11 (2022): e0277105. 36322585 10.1371/journal.pone.0277105 PMC9629630

[15] S. Matsumoto , H. Kawahira , K. Fukata , et al., “Laparoscopic Distal Gastrectomy Skill Evaluation From Video: A New Artificial Intelligence‐Based Instrument Identification System,” Scientific Reports 14, no. 1 (2024): 12432. 38816459 10.1038/s41598-024-63388-y PMC11139867

[16] H. Hwang , J. Lim , C. Kinnaird , et al., “Correlating Motor Performance With Surgical Error in Laparoscopic Cholecystectomy,” Surgical Endoscopy 20, no. 4 (2006): 651–655. 16391955 10.1007/s00464-005-0370-8

[17] T. Flash and N. Hogan , “The Coordination of Arm Movements: An Experimentally Confirmed Mathematical Model,” Journal of Neuroscience 5, no. 7 (1985): 1688–1703. 4020415 10.1523/JNEUROSCI.05-07-01688.1985 PMC6565116

