# Supplementary figures and images for: Evaluation of Experienced Manipulation Characteristics in Robotic Surgery Using Log Data From the Hinotori Surgical System
Source: Ann Gastroenterol Surg. 2026 Jan 30;10(4):1320–30. doi: 10.1002/ags3.70189 (PMC13327084; doi:10.1002/ags3.70189)

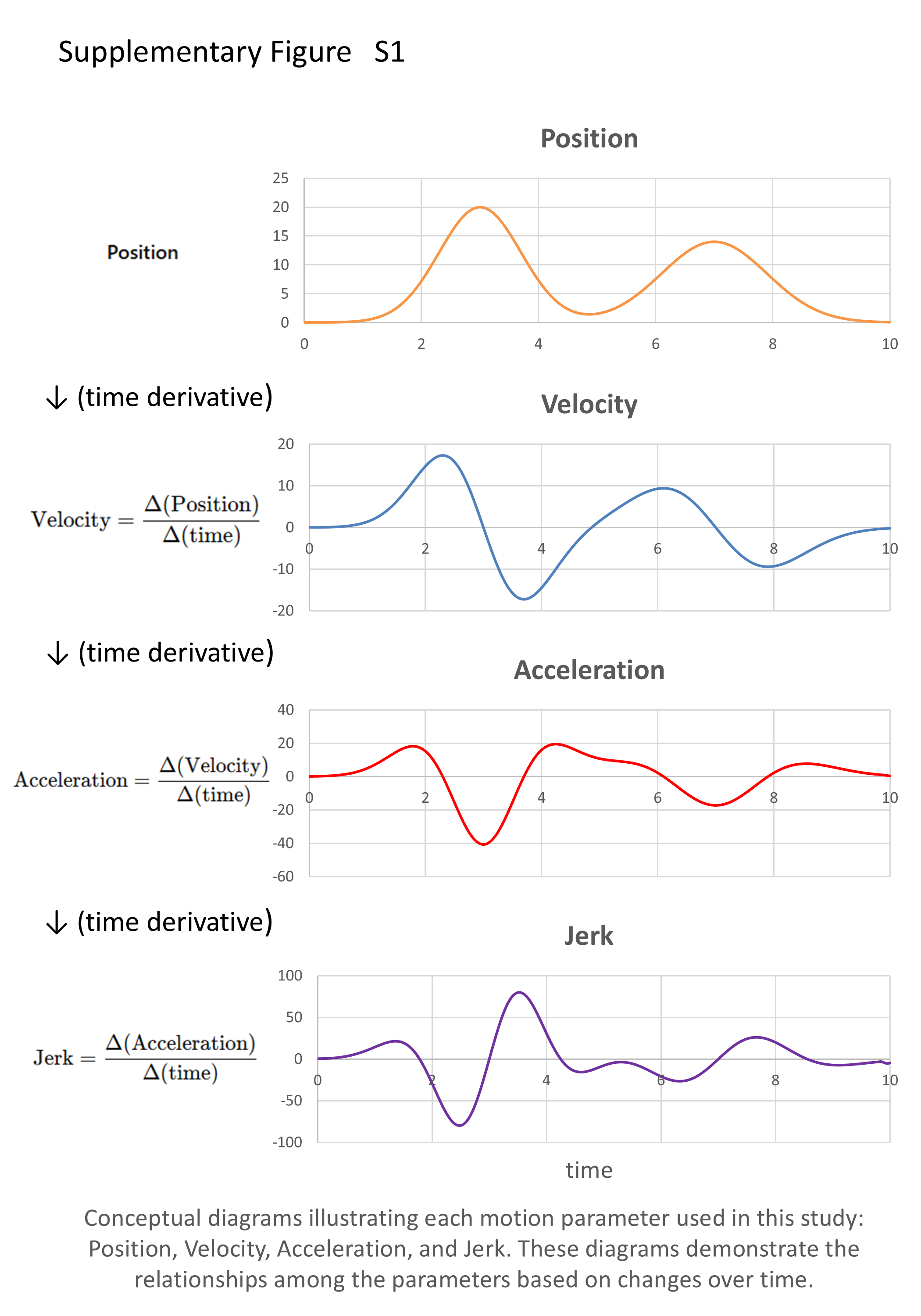

Supplement: Supplementary file 1 — Figure S1: A conceptual diagram illustrating the relationships among Position, Velocity, Acceleration, and Jerk. [file AGS3-10-1320-s004.tif]

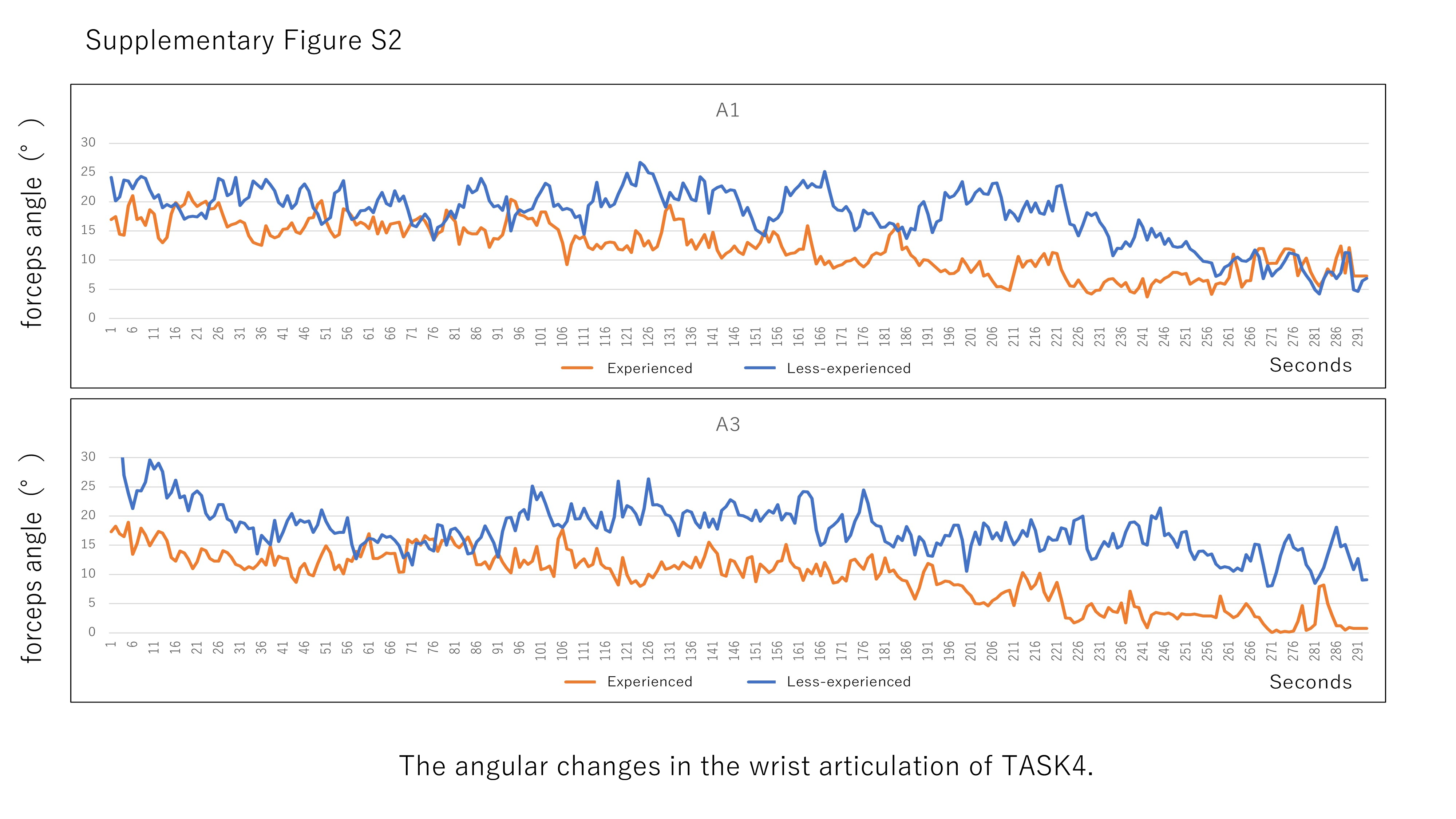

Supplement: Supplementary file 2 — Figure S2: Angular changes in wrist articulation during Task 4. [file AGS3-10-1320-s001.tif]

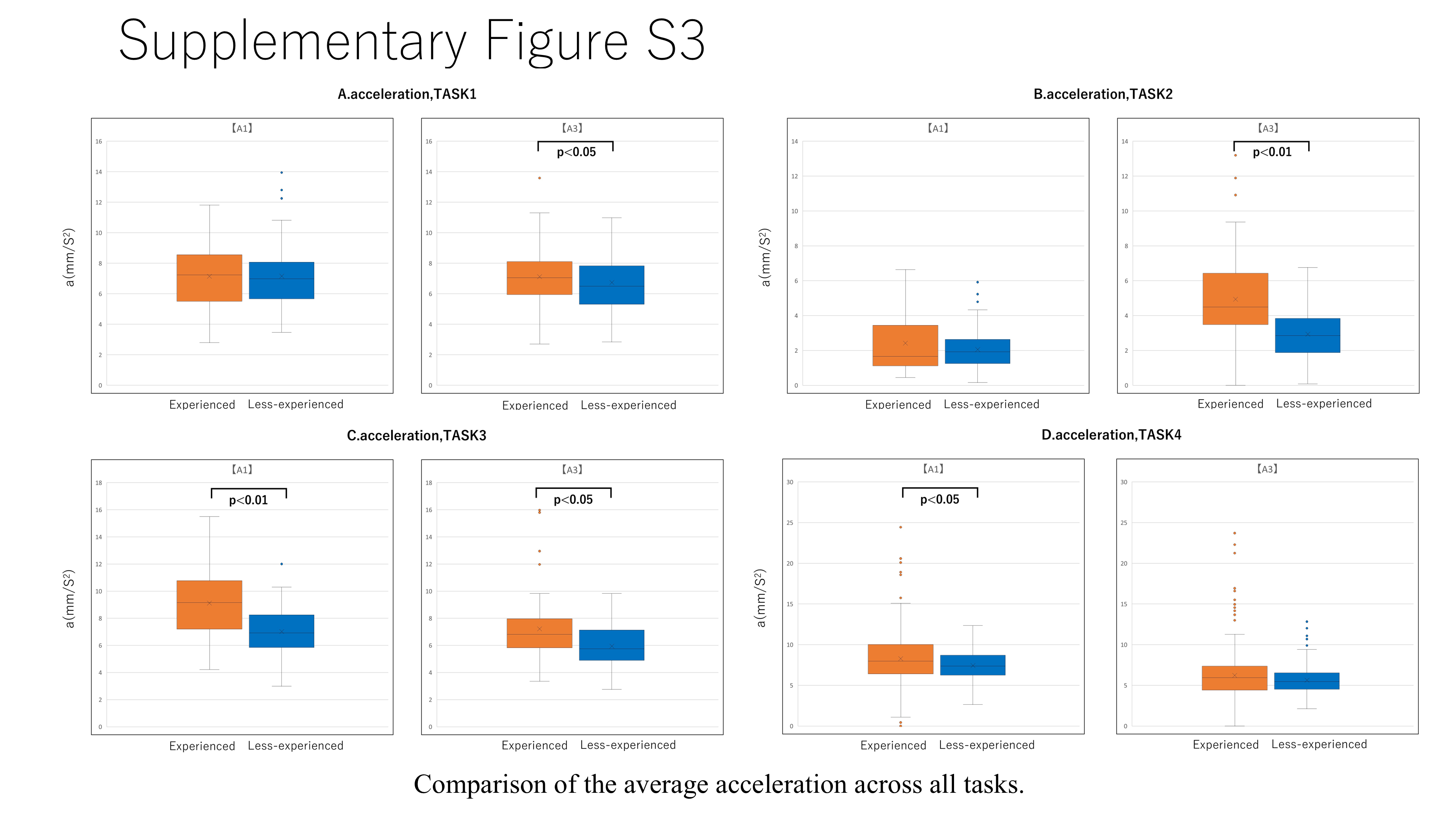

Supplement: Supplementary file 3 — Figure S3: Comparison of average acceleration across all tasks. [file AGS3-10-1320-s006.tif]
