# Supplementary material for: Evaluation of Experienced Manipulation Characteristics in Robotic Surgery Using Log Data From the Hinotori Surgical System
Source: Ann Gastroenterol Surg. 2026 Jan 30;10(4):1320–30. doi: 10.1002/ags3.70189 (PMC13327084; doi:10.1002/ags3.70189)
Supplement: Supplementary file 4 — Table S1: Background characteristics of E and L groups. [file AGS3-10-1320-s008.docx]

Supplementary Table S1

Background characteristics of E and N groups

|  | E group  (n = 6) | N group  (n = 6) | *p* value |
| --- | --- | --- | --- |
| Years of surgical experience (years) | 23.5 (21.5, 26.3) | 8.5 (6.5, 12.0) | 0.004 |
| Gender (Male/Female) | 6/0 | 6/0 | - |
| Dominant hand (R/L) | 6/0 | 5/1 | 1.000 |
| Primary robotic surgery experience (Yes/No) | 6/0 | 5/1 | 1.000 |

Median and interquartile range (IQR; 25%–75%)
